# Physics-assisted machine learning methods for predicting the splitting tensile strength of recycled aggregate concrete

**DOI:** 10.1038/s41598-023-36303-0

**Published:** 2023-06-05

**Authors:** Jianguo Liu, Xiangyu Han, Yin Pan, Kai Cui, Qinghua Xiao

**Affiliations:** School of Civil Engineering, Southwest Jiaotong University, Chengdu, 610031 People’s Republic of China

**Keywords:** Civil engineering, Mechanical properties

## Abstract

Recycled aggregate concrete (RAC) has become a popular building material due to its eco-friendly features, but the difficulty in predicting the crack resistance of RAC is increasingly impeding its application. In this study, splitting tensile strength is adopted to describe the crack resistance ability of RAC, and physics-assisted machine learning (ML) methods are used to construct the predictive models for the splitting tensile strength of RAC. The results show that the AdaBoost model has excellent predictive performance with the help of the Firefly algorithm, and physical assistance plays a remarkable role in selecting features and verifying the ML models. Due to the limit in data size and the generalizability of the model, the dataset should be supplemented with more representative data, and an algorithm for small sample sizes could be studied in the future.

## Introduction

According to reports from the National Development and Reform Commission of China, the total production of commercial concrete exceeded 32 billion cubic meters in 2021^1^. Meanwhile, with the demolition of old and abandoned buildings, more than 2 billion tons of concrete waste has been produced. Hence, recycled aggregate concrete (RAC), which uses recycled concrete waste to replace some coarse aggregates, has become a new trend in concrete production. Normally, the cost of using recycled aggregate in concrete is generally lower than that of using new materials. RAC also contributes to reducing the amount of waste that goes to landfills as well as the amounts of energy and carbon emissions needed to produce new materials.

**Figure 1:**
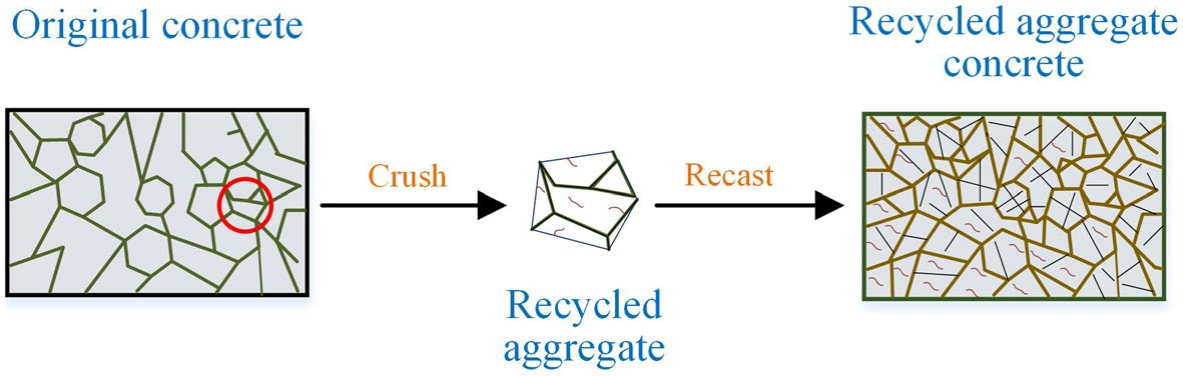
Various weak regions of recycled aggregate concrete.

During the recycled aggregate manufacturing process, the obtained recycled aggregate always consists of natural aggregate and hardened mortar. Hence, compared with that of natural aggregate, the recycled aggregate is weaker due to the weakness of hardened mortar and the interfaces between the mortar and natural aggregate. Furthermore, a large number of cracks are generated inside the recycled aggregate during the crushing process^2,3^. As shown in Fig. 1, various weak regions are introduced into recycled aggregate concrete, and such material is peculiarly prone to cracking when exposed to external loads, which severely restricts its application. Hence, compared with other mechanical properties, the cracking properties of RAC deserve more attention.

The presence of various fibers improves the ability of fiber-reinforced RAC to resist cracking^4–6^. Akca et al.^7^ applied polypropylene fibers to reinforce RAC and proved that both flexural tensile strength and splitting tensile strength increase with increasing fiber content. Ali et al.^8^ compared the mechanical properties of glass fiber-reinforced RAC and plain RAC, and an obvious increase was witnessed in the splitting tensile strength of fiber-reinforced recycled aggregate concrete. Gao et al.^9^ studied the performance of steel fiber-reinforced RAC, and the flexural strength was improved significantly with increasing steel fiber volume fraction. It can be concluded that the fibers could efficiently improve the cracking resistance of RAC, but the mechanism of reinforcement is affected by various factors.

For plain concrete, various methods have been proposed to predict its cracking performance, such as fracture tests, numerical methods and mechanical models^10–13^, and different factors are studied to guarantee the prediction reliability. Even so, it is still difficult to precisely describe the cracking characteristics of concrete. Considering the influence of recycled aggregate, the prediction of the cracking behavior of RAC, especially of RAC with fibers, is more complex. Machine learning methods are novel approaches for prediction issues^14–16^. Pan and Amin attempted to use machine learning (ML) to predict the cracking characteristics of RAC^17,18^ and established several ML models with superior performance. However, during the construction of ML models, the physical meaning of the model is neglected, and the influence of fibers is not considered.

**Figure 2:**
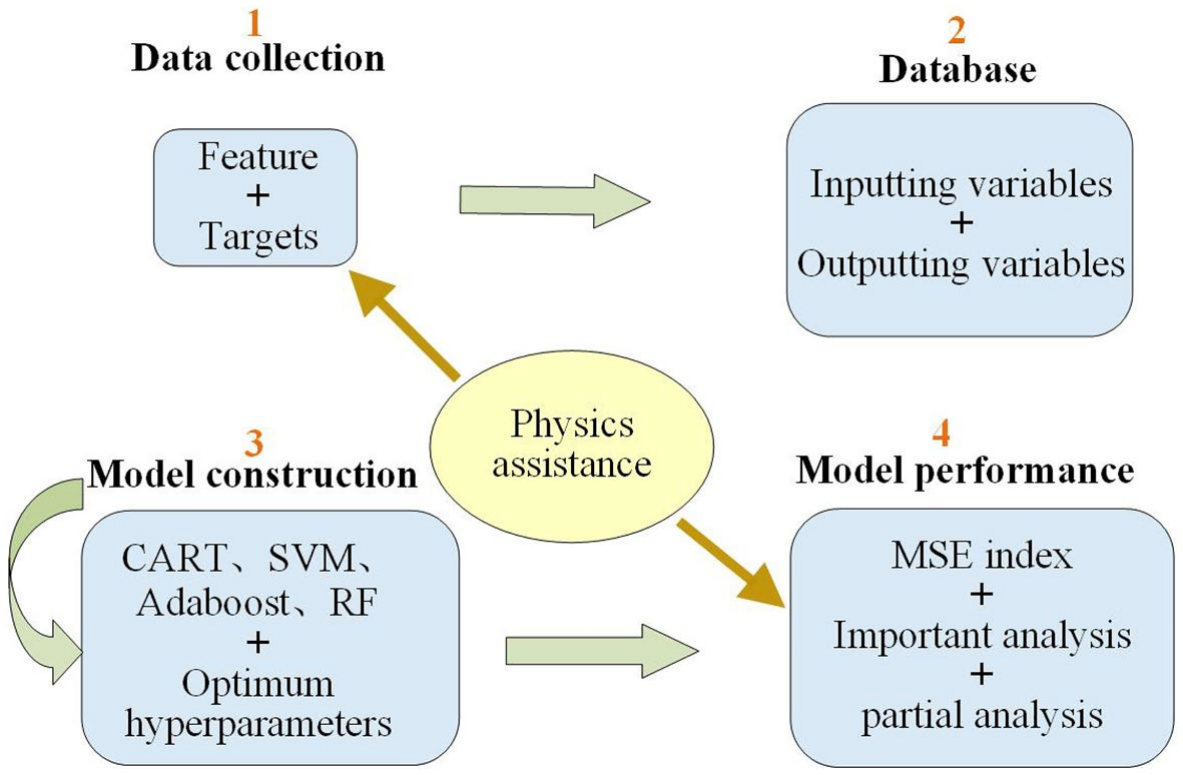
Flowchart of the physics-assisted machine learning process in this study.

In this study, machine learning approaches are combined with existing mechanical models and physical experiments, and a precise prediction of the splitting tensile strength of RAC is attempted. This paper is arranged as shown in Fig. 2 and includes (1) the construction of a database with the help of physical theories, (2) the illustration of several machine learning algorithms used in the current study, (3) the presentation of the construction process of predictive models, (4) the testing of the performance of predictive models with mathematical and physical methods and (5) the presentation of conclusions.

## Methods

### Physics-assisted construction of the database

For concrete materials, a splitting test is always adopted to observe their cracking behavior, and the splitting tensile strength is applied to evaluate their ability to resist cracking. Hence, the splitting tensile strength of RAC is selected as the predicted target (output variables) to represent the cracking performance, and various influencing factors are regarded as the input variables. These two types of variables compromise machine learning databases.

As the first step of machine learning, the selection of variables and collection of data are significantly important, which affects the prediction accuracy and generalizability of ML models. For conventional ML models, the physical meaning of variables is always neglected, leading to blind ML models that cannot handle cases outside of the constructed database. Therefore, physical experiments and existing mechanical models are used to assist in the construction of the database.

Since there are no well-established mechanical models for RAC, plain concrete cracking models are referenced here. According to well-known mechanical models, such as the fictitious-crack model, size effect model, and boundary effect model, the cracking/fracture properties of concrete are irrelevant to the specimen geometries but determined by mixture design^13,19,20^. Hence, the contents of water, cement and coarse aggregate and the size of aggregate are chosen as the input variables. Moreover, based on published studies^4,6,21–23^, the splitting tensile strength is also affected by other factors, such as the characteristics of the recycled aggregate (content, density, water absorption, size), the fiber types and the volume fractions.

Based on the established guidance for selecting features, a total of 257 data points were collected from the published literature^2,4–6,8,9,21–44^. It should be noted that in these collected experiments, the testing process follows the requirements of relevant standards, such as the Standard Test Method for Splitting Tensile Strength of Cylindrical Concrete Specimens (ASTM C496/C496M-2017) and the Standard for Test Method of Mechanical Properties on Ordinary Concrete (GB/T 20081-2002), and the obtained splitting tensile strength can be regarded as the material property. In most experiments on RAC, to eliminate the entropy factor in the results, natural and recycled aggregates are always sieved together and have the same size distribution, and the sizes of natural and recycled aggregates are incorporated into the index of the maximum size of coarse aggregates. Consequently, the mathematical characteristics of 10 numeric variables and one nonnumeric variable are listed in Table 1. These include water, cement, NCA (natural concrete aggregate) content, RCA (recycled concrete aggregate) content, SP (superplasticizer), D_max__RCA (maximum aggregate size of RCA), *ρ*_*RCA*_ (density of RCA), W_RCA_ (water absorption of RCA), fibers, STS (splitting tensile strength) and fiber type. A scattered distribution can be found in these numeric variables, and their values are quite different. Hence, to guarantee that the values of each feature can be reasonably used during the training process, preprocessing work is implemented for these data. First, the missing values are treated with K-nearest neighbor (KNN) methods. Then, to ensure the equal status of different features, min–max normalization is applied to these variables, as shown in Eq. (1), where *x*_*min*_ and *x*_*max*_ are the minimum and maximum values of feature *x,* respectively.

1 $$ x_{i}^{\prime } = \frac{{x_{i} - x_{\min } }}{{x_{\max } - x_{\min } }}(i = 1,2,3 \ldots n) $$

**Table 1:** The characteristics of the collected data.

Item	Mean	Minimum	Maximum	Standard deviation
Water (kg/m^3^)	185.6	98.3	343.5	37.4
Cement (kg/m^3^)	369.9	158.0	600.0	62.0
NCA (kg/m^3^)	344.8	0	1143.0	363.6
RCA (kg/m^3^)	732.8	57.0	1474.0	387.4
SP (kg/m^3^)	1.0	0	7.8	1.6
D_max__RCA (mm)	18.1	10.0	25.0	4.3 k
*ρ*_*RCA*_ (kg/m^3^)	2431.0	2010.0	2661.0	156.7
W_RCA_ (%)	5.4	1.9	10.9	1.8
Fibers (vol. %)	0.3	0	2.0	0.4
STS (MPa)	3.1	1.4	7.6	1.1
Fiber type	Steel fiber, Carbon fiber, Polypropylene fiber, Basalt fiber, Glass fiber, Woolen fiber			

**Figure 3:**
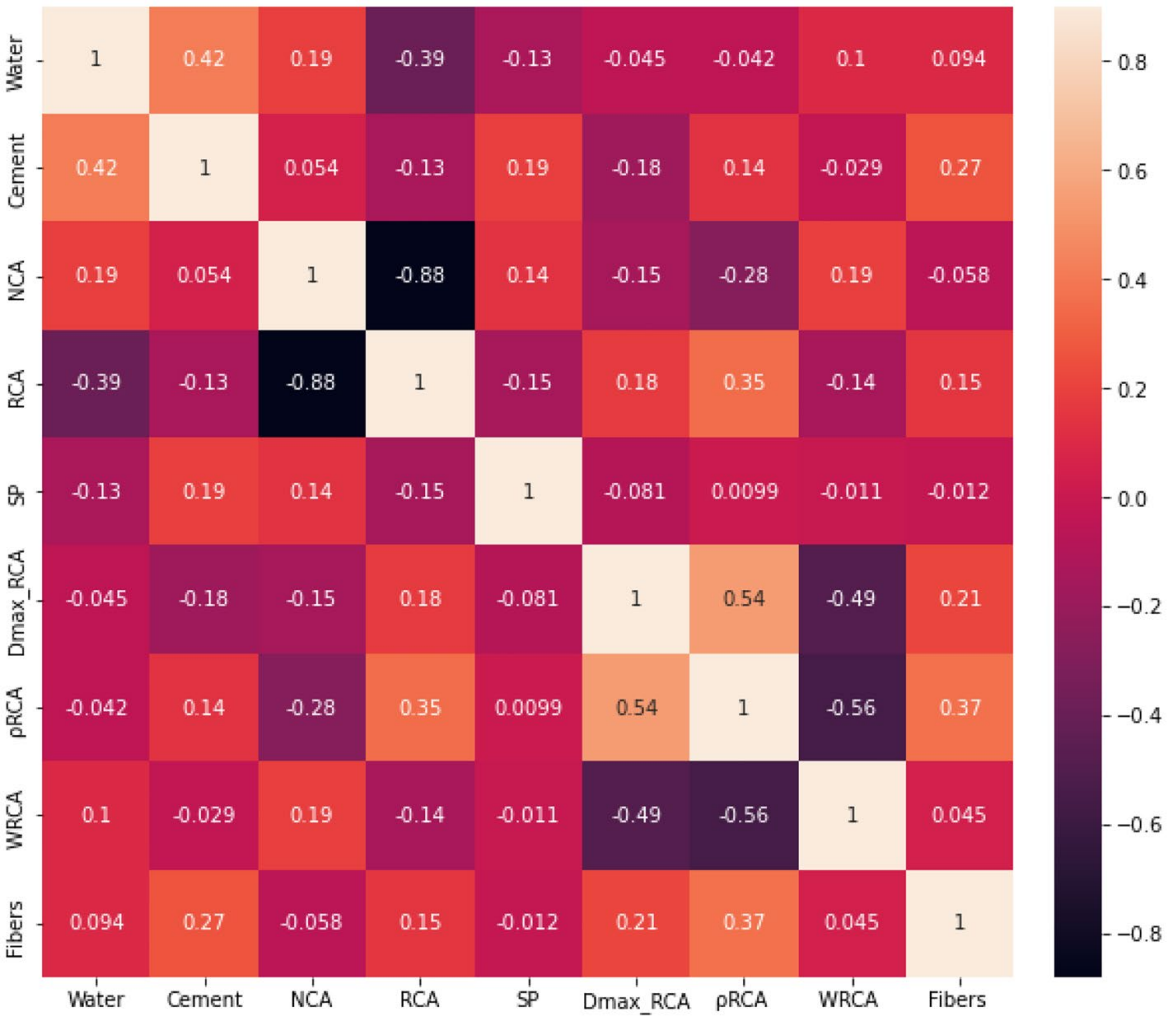
Correlation analysis of 9 numeric input variables.

Next, since the fiber type value is nonnumeric and cannot be directly used in the training or testing process, these values are converted into numeric values. Finally, the correlation between 9 numeric input variables is analyzed with a correlation matrix, as shown in Fig. 3. There is a strong relationship between the NCA and RCA content. From the physics perspective, to ensure the workability and mechanical properties of recycled aggregate concrete, the total contents of coarse aggregate are determined using a mixture design, and the NCA and RCA contents are directly related. Hence, the features of NCA are dropped here. Consequently, 8 numeric features and 1 nonnumeric feature are identified as the input variables, and the splitting tensile strength is regarded as the output variable.

### Machine learning algorithms

1. Classification and regression tree (CART) As the most frequently used supervised machine learning method, the classification and regression tree (CART) algorithm can be easily applied in classification and regression problems^45^. The difference between a classification tree and a regression tree is the type of target values. When the target belongs to a discrete variable, the CART is a classification tree. When the target belongs to a consecutive variable, the CART is a regression tree. In this study, the CART is a regression tree.

**Figure 4:**
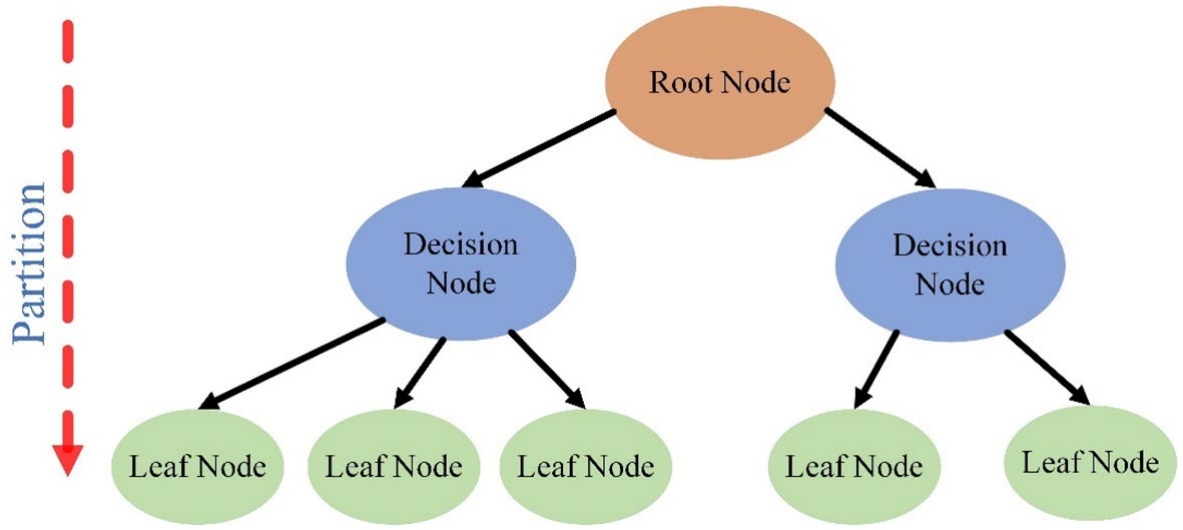
The schematic of CART.

A conventional CART is composed of a root node, a decision node and a leaf node, as shown in Fig. 4. The root node that contains all the data is split into two subsets following the recursive binary splitting criterion. During the splitting procedure, the data in each subset should be kept as homogeneous as possible. Then, the decision node is split into two sets following the same principle, and the complexity of the variance in each subset is reduced, but the model becomes increasingly complicated. Such a partitioning process will stop when one of the following conditions is met: (1) when the data in each leaf node share the same characteristics or (2) when the depth of the tree reaches its maximum value. Consequently, a fully grown tree is generated.

For a fully grown tree, a predictive model often has excellent performance during the training process but cannot precisely predict the target values with the testing dataset. This overfitting phenomenon is caused by the complex structure of CART. Hence, another essential procedure of the CART algorithm is pruning work. First, redundant branches are removed from the bottom of the fully grown tree, forming a sequence that consists of different subtrees. Then, the cross-validation method is used to test the performance of subtrees and select the optimum subtree as the ultimate predictive model. During the training of each CART model, the following parameters should be determined: max_depth, min_samples_split, and min_samples_leaf.

2. Support vector regression (SVR).

Support vector regression (SVR) is a kind of support vector machine algorithm that addresses regression problems^46^. Due to its high prediction accuracy and lower computational power requirements, SVR has been widely used in various fields.

**Figure 5:**
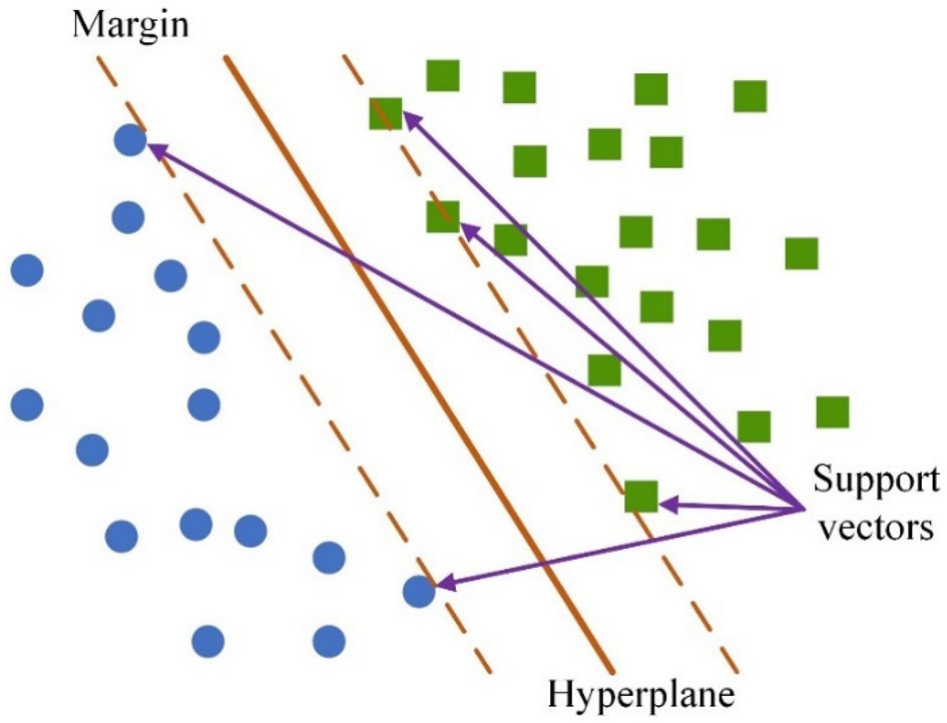
Schematic of the SVR algorithm.

The aim of SVR is to search a hyperplane that distinctly classifies the data points in the training data, and the dimensions of the hyperplane are determined by the number of features. There are various candidate hyperplanes, and the optimal plane often has the maximum distance between data points of both classes so that the data points can be partitioned with more confidence, as shown in Fig. 5. The data points that are close to the hyperplane are referred to as support vectors, and the position and orientation of the hyperplane are determined by these vectors. The two lines that are drawn around the hyperplane at a distance of *ε* are referred to as boundary lines, and they are used to create a margin between the data points. The value of *ε* reflects the tolerance of error in the training process, and a higher *ε* indicates a higher generalizability. Moreover, another essential part of SVR is the kernel, which consists of a set of mathematical functions. With the assistance of different kernels, the data can be transformed into the required form so that the hyperplane can be found in a higher dimensional space.

From the description of the SVR algorithm, it can be found that SVR is easy to implement and robust to outliers. However, the model will have poor performance when the dataset has too much noise. For one given SVR model, the C_penelty, kernel and tolerance should be determined during training.

3. Adaptive boosting (AdaBoost).

During the construction of machine learning models, it is essential to balance the prediction accuracy and generalizability of ML models. Prediction models with higher accuracy always have poor generalizability, and vice versa. Hence, ensemble learning algorithms are proposed to solve such problems.

**Figure 6:**
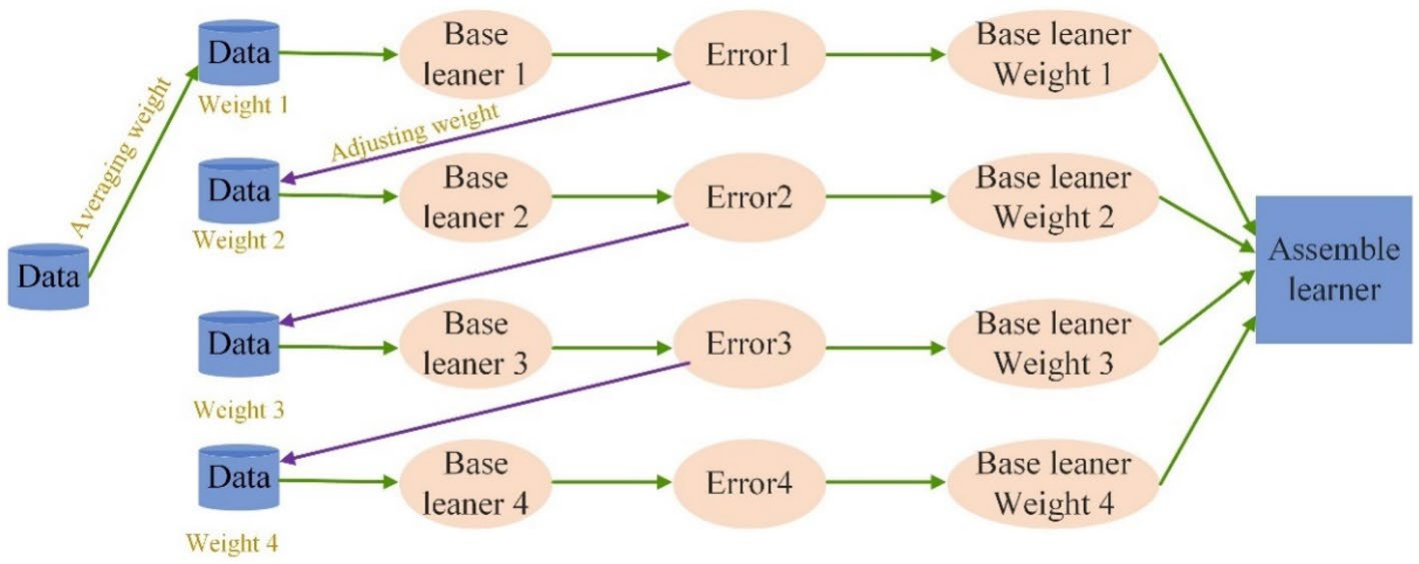
The procedure of the Adaboost algorithm.

The AdaBoost algorithm is a kind of ensemble learning algorithm that integrates various base learners^47^. As shown in Fig. 6, the procedure of Adaboost can be summarized as follows: (1) evenly assign the weights of all data points; (2) use the data to train the base learner and calculate its error; (3) adjust the weights of the data point by reducing the weights of accurately predicted data and increasing the weights of incorrectly predicted data; (4) repeat Step (2) and Step (3); and (5) integrate these base learners into the ensembled learner. The base learners with low error rates will have larger weights, and the base learners with high error rates will have smaller weights. Consequently, prediction models with good robustness are established. During construction of Adaboost models, the max_depth, min_samples_split, min_samples_leaf, n_estimators and loss are needed.

4. Random forest (RF).

The random forest (RF) algorithm is another important ensemble algorithm that was proposed by Breiman^48^. To improve the prediction accuracy and control overfitting, the bagging technique is applied to average the parallel base learners in RF.

**Figure 7:**
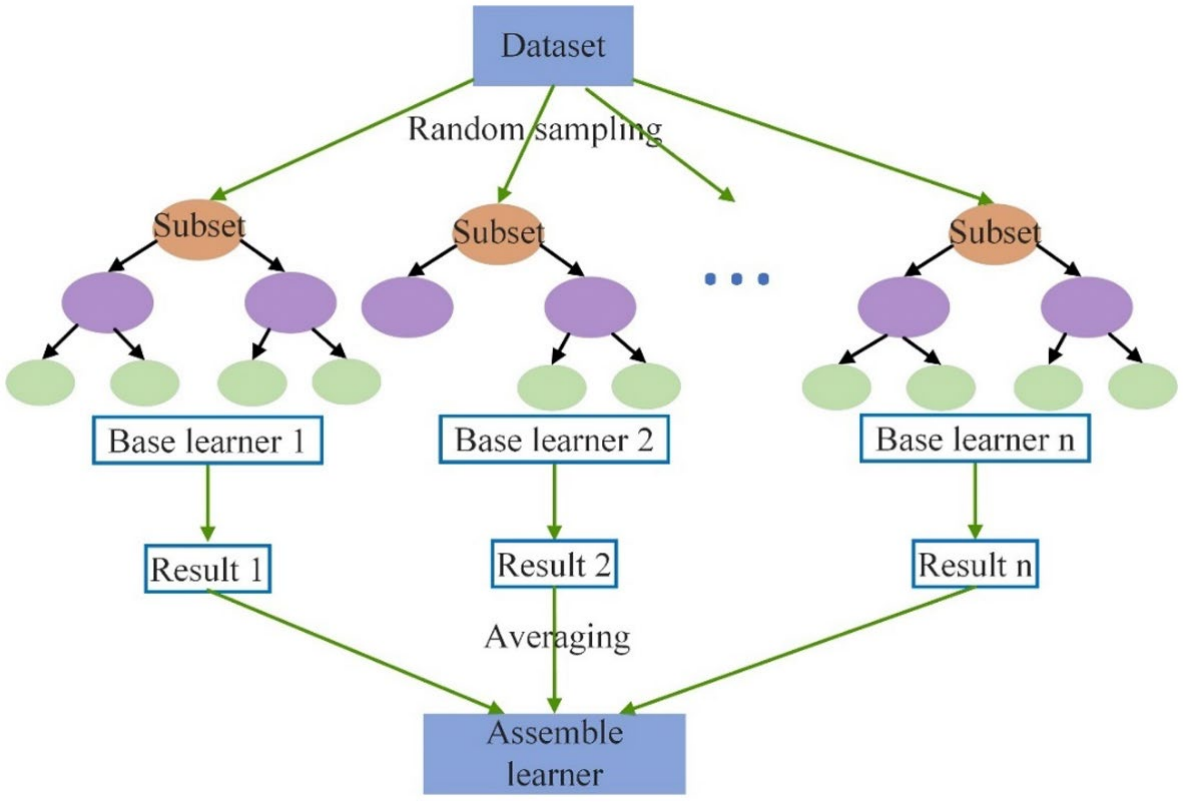
Flowchart of the random forest algorithm.

As shown in Fig. 7, the random forest algorithm consists of sampling, model training, predicting and averaging procedures. First, the data points are randomly selected to construct subsets with replacements, and overlaps exist between different subsets. Next, during the growth of trees, the attributes to split the nodes are randomly selected. Thus, the pruning process is not necessary here. Then, these isolated base learners are used to implement prediction work. Finally, for regression problems, the base learners are integrated by averaging the predictive results in each iteration. It should be noted that the randomness of RF is introduced by data sampling and node splitting, and all the base learners are independent. Moreover, the following parameters could be set for better RF performance, such as max_depth, min_samples_split, min_samples_leaf, and n_estimators.

### Construction of predictive models

As illustrated in the above section, a total of 257 data points are prepared in the current study. To train and test the predictive models, the dataset, which consists of input variables and output variables, should be split into two subsets, a training set and a testing set, and the ratio between the training set and testing set is set as 0.7:0.3. Namely, 179 cases are assigned to construct the predictive models, and the remaining 78 cases are used to evaluate the model performance. It should be noted that to guarantee the generalizability of the predictive models, all the data should be randomly shuffled before dataset splitting.

During the training process, the predictive models are always constructed in a complex form, leading to different performance during training and testing. Hence, K-fold cross-validation is proposed to solve such problems by introducing an extra validation procedure during training^49^. First, the training subset is evenly partitioned into K parts. Next, in the first iteration, the former K−1 parts are applied to search the parameters of the predictive models, and the remaining part is used to validate the constructed models and calculate the prediction accuracy. Then, the above procedure is repeated K times to guarantee that each part can be used as a training set K-1 times and a validation set one time. Finally, the average value of the accuracy score in each iteration is regarded as the performance index of the constructed models. Therefore, the predictive models can be more adaptive to the data out of the range of the training set, and the generalizability is also enhanced. Moreover, K-fold cross-validation is helpful for tasks with limited data. In this study, the value of K is set as 10.

There is a large difference between the performance of AI models with different hyperparameters. Hence, another essential task during the training process is to search for the optimum hyperparameters. In this study, the firefly algorithm (FA) is applied to tune the hyperparameters of the prediction models. As a heuristic algorithm, the FA is inspired by the flashing behavior of fireflies^47^. The following conditions are assumed for FA: (1) fireflies are neutral with respect to sex, and these fireflies will be attracted to each other; (2) the attractiveness between fireflies is proportionate to their brightness, which is determined by the distance between them; and (3) if there is no brighter firefly than a given firefly, it will move randomly. After several iterations, the brightest firefly will be found. For the current predictive models, the mean squared error (MSE) is regarded as the objective function, as shown in Eq. (2), where $y_{i}$ and $\hat{y}_{i}$ are experimental and predicted values, respectively. When the MSE reaches the lowest value during tenfold cross-validation, the optimum hyperparameters are obtained.

2 $$ MSE = \frac{1}{n}\sum\limits_{i = 1}^{n} {(y_{i} - \hat{y}_{i} )^{2} } (i = 1,2,3 \ldots n) $$

**Figure 8:**
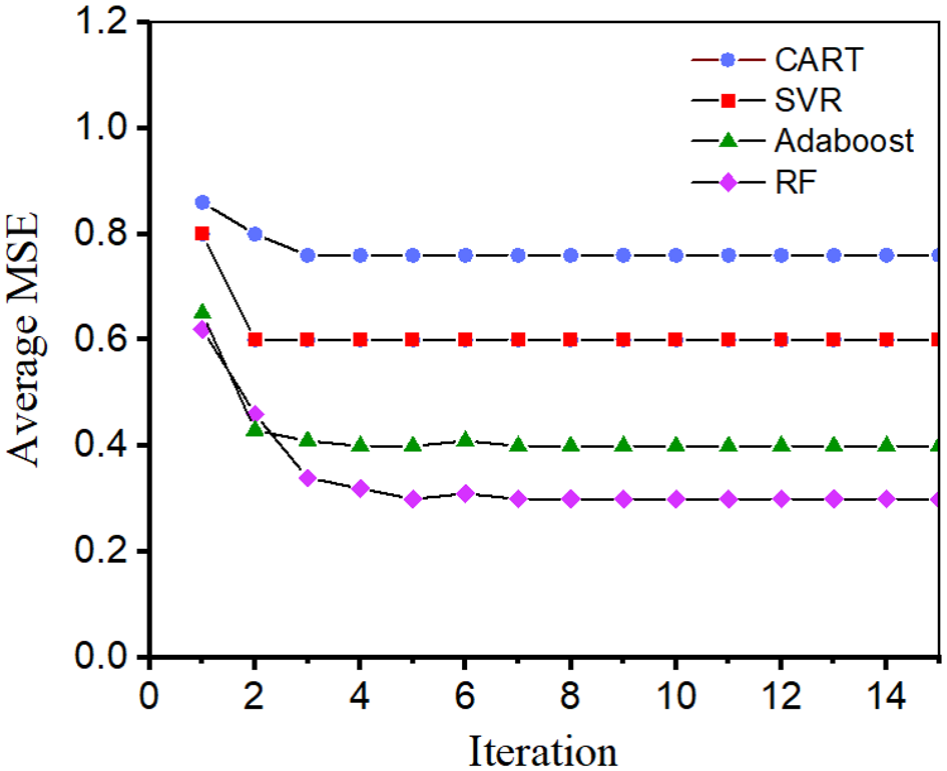
The variation in the average MSE with tuning iterations.

Therefore, ten-fold cross-validation and the FA algorithm are used to tune the hyperparameters of the 4 algorithms in section “Physics-assisted verification of prediction models”. As shown in Fig. 8, the MSE values of all the models reach a stable state within 6 iterations. That is, the FA algorithm is efficient for tuning the hyperparameters of the prediction models. Moreover, the RF and Adaboost models have better performance than that of the CART and SVR models. The optimum hyperparameters of the 4 models are listed in Table 2.

**Table 2:** The optimum hyperparameters of 4 AI models.

AI models	Hyperparameters	Optimum value
CART	max_depth	11
	min_samples_split	7
	min_samples_leaf	6
SVR	C_penelty	7.9
	kernel	linear
	tolerance	0.0001
Adaboost	max_depth	12
	min_samples_split	8
	min_samples_leaf	4
	n_estimators	323
	loss	exponential
RF	max_depth	19
	min_samples_split	3
	min_samples_leaf	2
	n_estimators	126

After the AI models are established, the following step is to test the generalizability of these models. As illustrated in above sections, the RF predictive model obtained the optimum performance during training. However, a qualified AI model should not only perform well in the training process but also have excellent performance in predicting unseen data.

Therefore, the 78 cases in the testing set are used to test the above 4 predictive models. First, the input variables are used to feed the AI models. Then, the predicted targets are obtained for each case. Then, the predicted targets are compared with the output variables. For regression problems, the difference between the predicted targets and real targets can evaluated with the MSE score.

**Figure 9:**
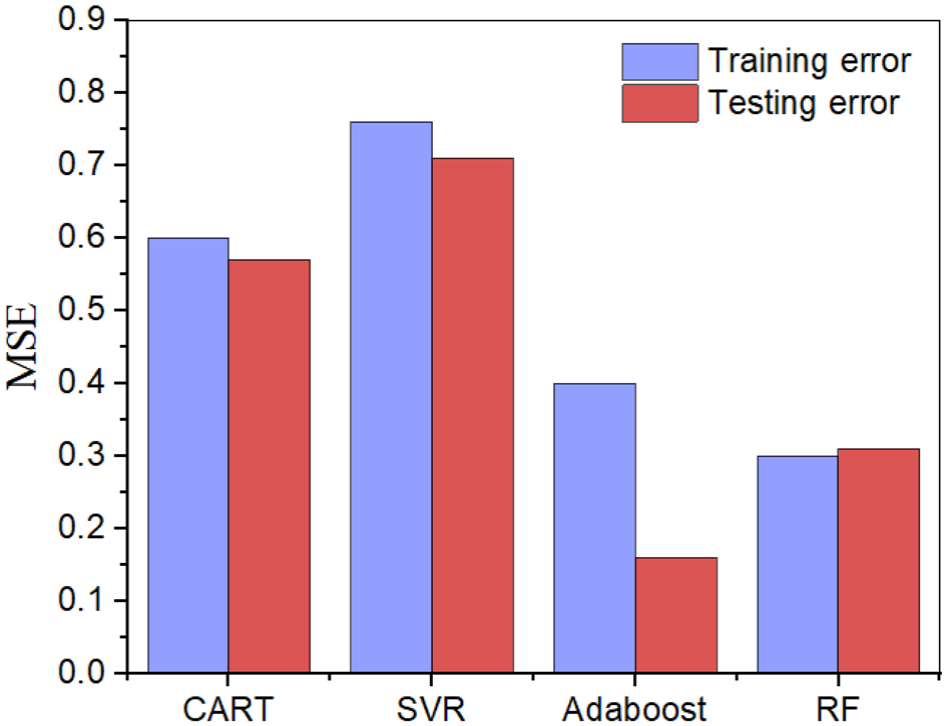
Comparison of the prediction performance between four AI models.

**Figure 10:**
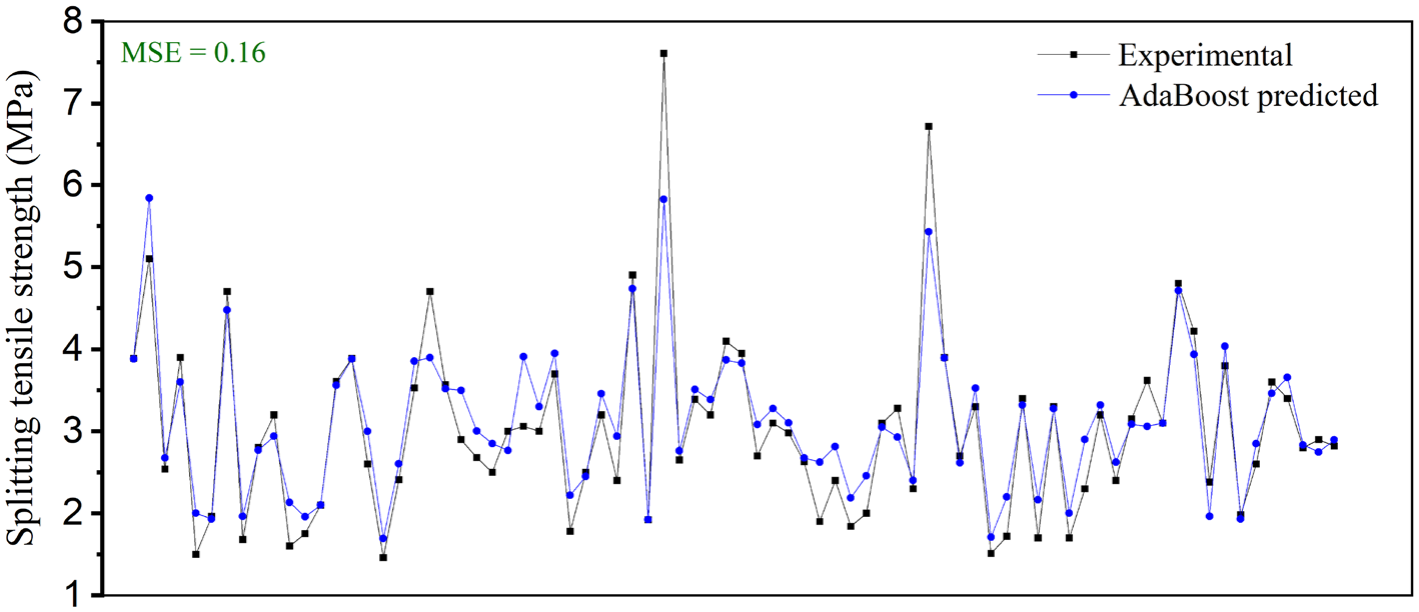
Comparison between the experimental and AdaBoost-predicted splitting tensile strength values.

As shown in Fig. 9, the performance of the four AI models is compared. The ensemble models have better performance than that of the CART and SVM models in both the training and testing procedures. By integrating several different base learners, the prediction accuracy and generalizability of ensembled models are enhanced. Although the MSE of the AdaBoost model is slightly higher than that of the RF model in the training procedure, the AdaBoost model has better performance in the testing process. Then, the predicted values using AdaBoost and the real values are compared, as shown in Fig. 10, and the high consistency of the results reflects the excellent prediction ability of the AdaBoost model.

## Discussion

### Physics-assisted verification of prediction models

Normally, AI models are regarded as a black box. These models pay much more attention to the reliability of the data than the physical meaning of the parameters. Consequently, the constitution of AI models is always too complex to interpret. Hence, the importance of features is proposed as an index to evaluate the contribution of various features to the AI model.

**Figure 11:**
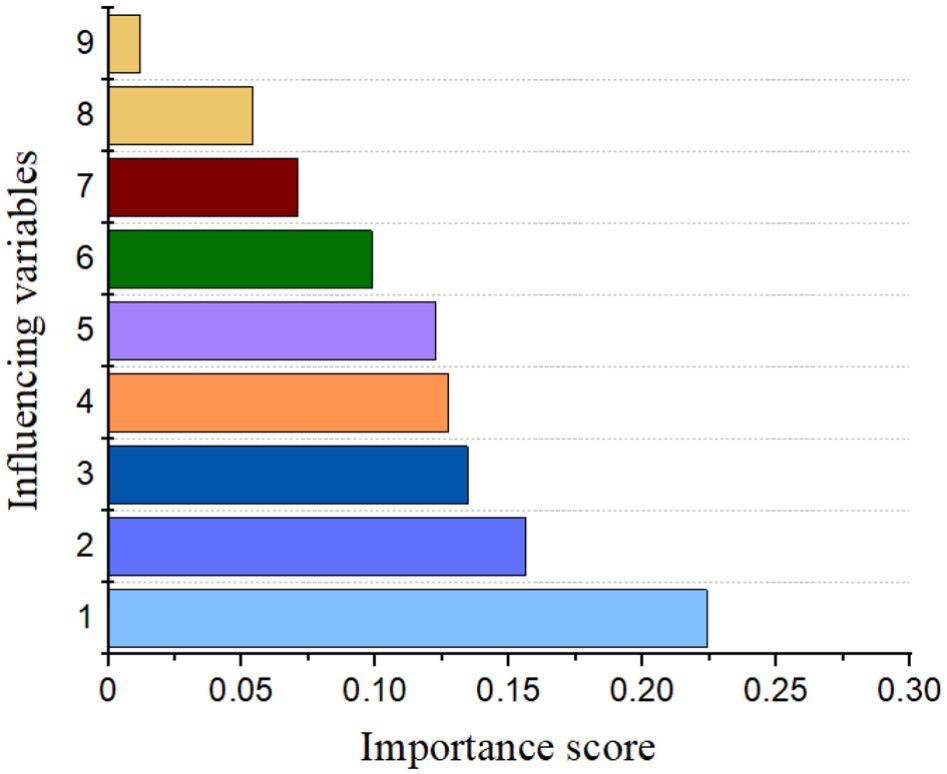
The importance of various influencing variables.

In this section, the AdaBoost model is selected to analyze the importance of 9 features due to its excellent prediction performance. For each feature, its contribution to the impurity of the base learner of the AdaBoost model can be calculated. Then, by averaging the calculated values and implementing normalization, the importance score of each of the 9 features is obtained. As shown in Fig. 11, the importance of these features is ranked. The recycled aggregate content has the highest importance score of 0.23. The size of the recycled aggregate and water content also have considerable influence on the AI models. The fiber type is regarded as a negligible feature for the predictive models of the splitting tensile strength of RAC, which obtains the lowest importance score of 0.02.

After that, the predicted model of splitting tensile strength is explained from the perspective of physics. As illustrated above, the recycled aggregate content, recycled aggregate size and water content are regarded as the three most influential factors. For a given RAC mixture, an increase in the recycled aggregate content will lead to a reduction in the natural aggregate content. Due to the inner voids and cracks, the RAC always fractures around these weak regions, which will further impair the splitting tensile strength of RAC. Moreover, when incorporating recycled aggregates, the contents of ITZs (interfacial transition zones) are enhanced, which will introduce a weak RAC location. Hence, the recycled aggregate content has a significant impact on the splitting tensile strength of RAC, and this conclusion is consistent with that of other AI models^18^. Next, the influence of the maximum aggregate size is analyzed. In the collected literature, the maximum size of recycled aggregate and natural aggregate is always consistent, and the maximum aggregate size index indicates the recycled aggregate and natural aggregate. In the splitting process of RAC, a crack will propagate along the surface of the natural aggregate or cross the recycled aggregate. Hence, the aggregate size will determine the cracking path and further affect the splitting strength. Then, the water and cement contents, which determine the w/c ratios, have been proven to be relevant to the tensile strength of various concretes. Finally, the fiber volume and fiber types are also regarded as influential factors for the splitting tensile strength of RAC but are not fully reflected in the AI models. This phenomenon may be caused by the distribution of collected data.

Then, the AI models are verified by physical experiments. From the importance analysis of different features, the recycled aggregate content is proven to be the most influential feature for the AI predictive models. Then, the dependence of AI models on these features is analyzed using partial dependence analysis.

The partial dependence analysis can be implemented as follows: (1) select one feature as the research object, (2) change the object in a reasonable range, keeping other features unchanged, (3) feed the AI models with different input variables, obtaining the predictive targets, (4) analyze the dependence of the AI models on the selected feature, and (5) compare the variation tendency with the experimental results.

**Figure 12:**
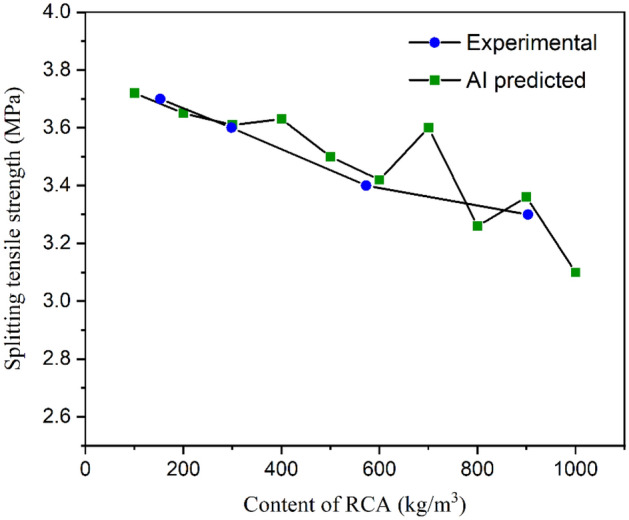
Variation in splitting tensile strength with the change in RCA content.

Taking the experiments in Reference^18^ as an example, the recycled aggregate content varies from 100 to 1000 kg/m^3^, and the other 8 features are kept consistent with the mixture design in the experiments. These features are set as the input variables, and the constructed AdaBoost models in section “Discussion” are used to predict the splitting tensile strength of RAC. Figure 12 illustrates the variation tendency of the AI-predicted splitting tensile strength with the change in recycled aggregate content, and the experimental results are also plotted in the same coordinates. The prediction accuracy of the splitting tensile strength is acceptable. An obvious reduction occurs in the splitting tensile strength when the recycled aggregate content increased from 100 to 1000 kg/m^3^, which was proven by physical experiments. Despite the visible deviation, the prediction of AI models is convincing.

Compared with that of previous research^17,18^, the construction of predictive models in the current study is convincing, and the established models are reliable with the assistance of physics. First, the influencing features are effectively selected from a physical view. Then, the feature importance analysis of predictive models is deeply discussed with mechanical theories, making the models more explainable. After that, partial dependence analysis of AI models is implemented with physical experiments, making the models more reliable.

## Conclusions

Due to various defects in recycled aggregates, the splitting tensile strength of RAC is weakened and interferes with the application of RAC. Fiber reinforcement methods have been proposed to solve such problems, but the cracking performance of these RACs is more complicated. In this study, four artificial intelligence methods are used to predict the tensile behavior of RAC, and physical assistance is used in the construction and verification of predictive models. The conclusions can be drawn as follows:

1. With the help of the FA, the optimum parameters of the models are efficiently searched. The mean square error (MSE) is selected to evaluate the performance of the model, and the MSE values for the CART, SVR, AdaBoost and RF models are 0.572, 0.713, 0.164 and 0.311, respectively. The superiority of the AdaBoost models can be attributed to the ensemble of various base learners.
2. Feature importance is determined, and the following input importance with an increasing pattern was observed for the AdaBoost models: recycled concrete aggregate (0.224) > maximum aggregate size (0.156) > water content (0.134) > cement content (0.127) > fiber superplasticizer (0.099) > density of RCA (0.071) > water absorption of RCA (0.054) > fiber type (0.012).
3. Physics is used to assist the machine learning models as follows: improving the efficiency of the construction of AI models by selecting highly related features, analyzing the feature importance from the fracture mechanism of RAC, and verifying the reliability of AI models with physical experiments.
4. The application of established predictive models can be described as follows: by collecting 9 features of one RAC and inputting these features into the models, the splitting tensile strength can be obtained. It should be noted that the physical verification of predictive results must be implemented to guarantee its reliability.

## Supplementary Information

Supplementary Information.

## Data Availability

All data generated or analysed during this study are included in the supplementary information files.
