# Supplementary material for: Physics-assisted machine learning methods for predicting the splitting tensile strength of recycled aggregate concrete
Source: Sci Rep. 2023 Jun 5;13:9078. doi: 10.1038/s41598-023-36303-0 (PMC10241791; doi:10.1038/s41598-023-36303-0)
Supplement: Supplementary file 1 — Supplementary Information. [file 41598_2023_36303_MOESM1_ESM.docx]

Table S1 Database for recycled aggregate concrete.

| Ref. | Water  (kg/m^3^) | Cement  (kg/m^3^) | NCA  (kg/m^3^) | RCA  (kg/m^3^) | SP  (kg/m^3^) | D_max___RCA_  (mm) | ρ_RCA_  (kg/m^3^) | W_RCA_  (%) | Fiber volume  (%) | Fiber types | STS  (MPa) |
| --- | --- | --- | --- | --- | --- | --- | --- | --- | --- | --- | --- |
| 1 | 207.6 | 400 | 863 | 153 | 0 | 20 | 2410 | 5.8 | 0 | 0 | 3.7 |
|  | 207.6 | 400 | 697 | 298 | 0 | 20 | 2410 | 5.8 | 0 | 0 | 3.6 |
|  | 207.6 | 400 | 383 | 573 | 0 | 20 | 2410 | 5.8 | 0 | 0 | 3.4 |
|  | 207.6 | 400 | 0 | 903 | 0 | 20 | 2410 | 5.8 | 0 | 0 | 3.3 |
| 2 | 165 | 300 | 905 | 267 | 4.98 | 25 | 2430 | 4.4 | 0 | 0 | 3 |
|  | 165 | 318 | 608 | 537 | 6.042 | 25 | 2430 | 4.4 | 0 | 0 | 3.2 |
|  | 162 | 325 | 0 | 1123 | 6.175 | 25 | 2430 | 4.4 | 0 | 0 | 3.2 |
| 3 | 180 | 400 | 886 | 215 | 0 | 10 | 2570 | 3.5 | 0 | 0 | 3.2 |
|  | 180 | 400 | 554 | 538 | 0 | 10 | 2570 | 3.5 | 0 | 0 | 3 |
|  | 180 | 400 | 0 | 1075 | 0 | 10 | 2570 | 3.5 | 0 | 0 | 2.8 |
|  | 225 | 410 | 840 | 204 | 0 | 10 | 2570 | 3.5 | 0 | 0 | 3.2 |
|  | 225 | 410 | 524 | 506 | 0 | 10 | 2570 | 3.5 | 0 | 0 | 3.2 |
|  | 225 | 410 | 0 | 1017 | 0 | 10 | 2570 | 3.5 | 0 | 0 | 3.1 |
| 4 | 225 | 410 | 840 | 204 | 0 | 10 | 2580 | 3.5 | 0 | 0 | 3.6 |
|  | 225 | 410 | 524 | 506 | 0 | 10 | 2580 | 3.5 | 0 | 0 | 3.4 |
|  | 225 | 410 | 0 | 1017 | 0 | 10 | 2580 | 3.5 | 0 | 0 | 3.3 |
|  | 205 | 410 | 865 | 210 | 0 | 10 | 2580 | 3.5 | 0 | 0 | 3.6 |
|  | 205 | 410 | 541 | 525 | 0 | 10 | 2580 | 3.5 | 0 | 0 | 3.6 |
|  | 205 | 410 | 0 | 1049 | 0 | 10 | 2580 | 3.5 | 0 | 0 | 3.4 |
|  | 180 | 400 | 886 | 215 | 5.6 | 10 | 2580 | 3.5 | 0 | 0 | 3.7 |
|  | 180 | 400 | 554 | 538 | 5.6 | 10 | 2580 | 3.5 | 0 | 0 | 3.7 |
|  | 180 | 400 | 0 | 1075 | 5.6 | 10 | 2580 | 3.5 | 0 | 0 | 3.5 |
|  | 160 | 400 | 912 | 221 | 7.8 | 10 | 2580 | 3.5 | 0 | 0 | 4.1 |
|  | 160 | 400 | 570 | 554 | 7.8 | 10 | 2580 | 3.5 | 0 | 0 | 4 |
|  | 160 | 400 | 0 | 1107 | 7.8 | 10 | 2580 | 3.5 | 0 | 0 | 3.8 |
| 5 | 175 | 350 | 711 | 297 | 1.68 | 25 | 2530 | 1.9 | 0 | 0 | 4.2 |
|  | 175 | 350 | 508 | 494 | 1.68 | 25 | 2530 | 1.9 | 0 | 0 | 4 |
|  | 175 | 350 | 0 | 989 | 1.68 | 25 | 2530 | 1.9 | 0 | 0 | 3.8 |
|  | 175 | 350 | 508 | 469 | 1.68 | 25 | 2400 | 6.2 | 0 | 0 | 3.4 |
|  | 175 | 350 | 0 | 938 | 1.68 | 25 | 2400 | 6.2 | 0 | 0 | 3.2 |
| 6 | 200 | 270 | 675 | 200 | 1.08 | 19 | 2440 | 5.8 | 0 | 0 | 1.9 |
|  | 210 | 270 | 450 | 400 | 1.35 | 19 | 2440 | 5.8 | 0 | 0 | 1.9 |
|  | 220 | 270 | 225 | 600 | 1.62 | 19 | 2440 | 5.8 | 0 | 0 | 1.4 |
|  | 165 | 370 | 760 | 230 | 1.48 | 19 | 2440 | 5.8 | 0 | 0 | 3.1 |
|  | 165 | 370 | 505 | 455 | 1.85 | 19 | 2440 | 5.8 | 0 | 0 | 3.1 |
|  | 165 | 370 | 250 | 680 | 2.59 | 19 | 2440 | 5.8 | 0 | 0 | 2.9 |
| 7 | 178.5 | 275 | 723.07 | 180.77 | 1.925 | 19 | 2400 | 5 | 0 | 0 | 2.4 |
|  | 178.5 | 275 | 423.77 | 423.77 | 1.925 | 19 | 2400 | 5 | 0 | 0 | 2.5 |
|  | 178.5 | 275 | 0 | 756.46 | 1.925 | 19 | 2400 | 5 | 0 | 0 | 2.6 |
|  | 190 | 380 | 7504 | 187.57 | 2.66 | 19 | 2400 | 5 | 0 | 0 | 3.1 |
|  | 190 | 380 | 443.71 | 443.71 | 2.66 | 19 | 2400 | 5 | 0 | 0 | 2.9 |
|  | 190 | 380 | 0 | 807.97 | 2.66 | 19 | 2400 | 5 | 0 | 0 | 2.9 |
| 8 | 172.43 | 401 | 911 | 303 | 0.2005 | 20 | 2661 | 1.9 | 0 | 0 | 2.3 |
|  | 172.43 | 401 | 585 | 585 | 0.70175 | 20 | 2602 | 2.6 | 0 | 0 | 2.1 |
|  | 172.43 | 401 | 0 | 1119 | 0.90225 | 20 | 2510 | 3.9 | 0 | 0 | 2 |
| 9 | 193 | 350 | 1061 | 57 | 0 | 12 | 2010 | 10.9 | 0 | 0 | 2.9 |
|  | 194 | 350 | 1061 | 170 | 0 | 12 | 2010 | 10.9 | 0 | 0 | 2.7 |
|  | 196 | 350 | 1061 | 283 | 0 | 12 | 2010 | 10.9 | 0 | 0 | 2.6 |
|  | 199 | 158 | 1061 | 566 | 0 | 12 | 2010 | 10.9 | 0 | 0 | 2.5 |
|  | 158 | 350 | 1111 | 59 | 3.5 | 12 | 2010 | 10.9 | 0 | 0 | 3.4 |
|  | 163 | 350 | 1105 | 177 | 3.5 | 12 | 2010 | 10.9 | 0 | 0 | 3.3 |
|  | 168 | 350 | 1100 | 294 | 3.5 | 12 | 2010 | 10.9 | 0 | 0 | 3.1 |
|  | 178 | 350 | 1089 | 582 | 3.5 | 12 | 2010 | 10.9 | 0 | 0 | 3 |
|  | 137 | 350 | 1143 | 61 | 3.5 | 12 | 2010 | 10.9 | 0 | 0 | 4.2 |
|  | 139 | 350 | 1143 | 183 | 3.5 | 12 | 2010 | 10.9 | 0 | 0 | 4.5 |
|  | 143 | 350 | 1138 | 304 | 3.5 | 12 | 2010 | 10.9 | 0 | 0 | 3.7 |
|  | 150 | 350 | 1132 | 605 | 3.5 | 12 | 2010 | 10.9 | 0 | 0 | 3.4 |
| 10 | 180 | 281 | 0 | 970 | 0 | 10 | 2360 | 4.7 | 0 | 0 | 3.5 |
|  | 170 | 293 | 0 | 919 | 0 | 10 | 2280 | 6.2 | 0 | 0 | 3.1 |
|  | 165 | 337 | 0 | 879 | 0 | 10 | 2220 | 7.8 | 0 | 0 | 3.3 |
|  | 190 | 463 | 0 | 970 | 0 | 10 | 2360 | 4.7 | 0 | 0 | 3.8 |
|  | 190 | 500 | 0 | 919 | 3.24 | 10 | 2280 | 6.2 | 0 | 0 | 3.7 |
|  | 180 | 600 | 0 | 879 | 5.04 | 10 | 2220 | 7.8 | 0 | 0 | 3.7 |
| 11 | 179 | 275 | 735 | 184 | 0 | 20 | 2320 | 5.3 | 0 | 0 | 2.8 |
|  | 179 | 275 | 455 | 455 | 0 | 20 | 2320 | 5.3 | 0 | 0 | 3.1 |
|  | 179 | 275 | 0 | 830 | 0 | 20 | 2320 | 5.3 | 0 | 0 | 2.4 |
|  | 190 | 380 | 757 | 189 | 0 | 20 | 2320 | 5.3 | 0 | 0 | 3.5 |
|  | 190 | 380 | 471 | 471 | 0 | 20 | 2320 | 5.3 | 0 | 0 | 2.7 |
|  | 190 | 380 | 0 | 874 | 0 | 20 | 2320 | 5.3 | 0 | 0 | 3.7 |
|  | 179 | 275 | 740 | 185 | 0 | 20 | 2320 | 5.3 | 0 | 0 | 2.5 |
|  | 179 | 275 | 408 | 408 | 0 | 20 | 2320 | 5.3 | 0 | 0 | 2.5 |
|  | 179 | 275 | 0 | 640 | 0 | 20 | 2320 | 5.3 | 0 | 0 | 2.3 |
|  | 190 | 380 | 767 | 192 | 0 | 20 | 2320 | 5.3 | 0 | 0 | 2.8 |
|  | 190 | 380 | 426 | 427 | 0 | 20 | 2320 | 5.3 | 0 | 0 | 2.6 |
|  | 190 | 380 | 0 | 683 | 0 | 20 | 2320 | 5.3 | 0 | 0 | 2.3 |
| 12 | 179 | 325 | 839 | 210 | 0 | 20 | 2320 | 5.3 | 0 | 0 | 2.8 |
|  | 179 | 325 | 490 | 490 | 0 | 20 | 2320 | 5.3 | 0 | 0 | 2.7 |
|  | 179 | 325 | 0 | 923 | 0 | 20 | 2320 | 5.3 | 0 | 0 | 2.3 |
|  | 173 | 385 | 892 | 223 | 0 | 20 | 2320 | 5.3 | 0 | 0 | 3.1 |
|  | 173 | 385 | 515 | 515 | 0 | 20 | 2320 | 5.3 | 0 | 0 | 3.9 |
| 13 | 146.5 | 380 | 543.2 | 505.1 | 0 | 10 | 2470 | 3.7 | 0 | 0 | 5. |
|  | 162.3 | 380 | 0 | 1010.2 | 0 | 10 | 2470 | 3.7 | 0 | 0 | 5.1 |
|  | 138.2 | 380 | 869.2 | 195 | 0 | 10 | 2390 | 4.9 | 0 | 0 | 6.3 |
|  | 149.8 | 380 | 543.2 | 487.5 | 0 | 10 | 2390 | 4.9 | 0 | 0 | 5.1 |
|  | 170.4 | 380 | 0 | 975.1 | 0 | 10 | 2390 | 4.9 | 0 | 0 | 5.9 |
|  | 139.7 | 380 | 869.2 | 187.8 | 0 | 10 | 2300 | 5.9 | 0 | 0 | 5.3 |
|  | 153.1 | 380 | 543.4 | 469.4 | 0 | 10 | 2300 | 5.9 | 0 | 0 | 6.2 |
|  | 175 | 380 | 0 | 938.8 | 0 | 10 | 2300 | 5.9 | 0 | 0 | 4.2 |
| 14 | 205 | 300 | 0 | 1075 | 0 | 20 | 2450 | 3.1 | 0 | 0 | 2.5 |
|  | 205 | 300 | 0 | 1027 | 0 | 20 | 2370 | 7.1 | 0 | 0 | 2.4 |
|  | 205 | 300 | 0 | 1027 | 0 | 20 | 2360 | 7.8 | 0 | 0 | 1.9 |
|  | 180 | 350 | 0 | 1089 | 0 | 20 | 2450 | 3.1 | 0 | 0 | 3.4 |
|  | 180 | 350 | 0 | 1041 | 0 | 20 | 2370 | 7.1 | 0 | 0 | 2.6 |
|  | 180 | 350 | 0 | 1041 | 0 | 20 | 2360 | 7.8 | 0 | 0 | 2.6 |
|  | 185 | 425 | 0 | 1028 | 0 | 20 | 2450 | 3.1 | 0 | 0 | 3.9 |
|  | 185 | 425 | 0 | 982 | 0 | 20 | 2370 | 7.1 | 0 | 0 | 3.7 |
|  | 185 | 425 | 0 | 982 | 0 | 20 | 2360 | 7.8 | 0 | 0 | 3.4 |
|  | 165 | 485 | 0 | 1039 | 0 | 20 | 2450 | 3.1 | 0 | 0 | 4.7 |
|  | 165 | 485 | 0 | 979 | 0 | 20 | 2370 | 7.1 | 0 | 0 | 4.1 |
|  | 165 | 485 | 0 | 982 | 0 | 20 | 2360 | 7.8 | 0 | 0 | 4.2 |
| 15 | 178.3 | 358 | 783.6 | 299.3 | 0.3 | 19 | 2570 | 2.7 | 0 | 0 | 3.9 |
|  | 178.3 | 358 | 458.3 | 598.4 | 0.3 | 19 | 2570 | 2.7 | 0 | 0 | 3.9 |
|  | 178.3 | 358 | 0 | 1020 | 0.3 | 19 | 2570 | 2.7 | 0 | 0 | 3.3 |
| 16 | 214.2 | 210 | 0 | 966 | 0 | 22 | 2451 | 7.8 | 0 | 0 | 2 |
|  | 196 | 280 | 0 | 940 | 0 | 22 | 2387 | 6.9 | 0 | 0 | 2.9 |
|  | 161 | 350 | 0 | 974 | 3.5 | 22 | 2362 | 4.2 | 0 | 0 | 4.6 |
|  | 212.1 | 210 | 0 | 970 | 0 | 22 | 2456 | 7.5 | 0 | 0 | 2 |
|  | 193.2 | 280 | 0 | 970 | 0 | 22 | 2455 | 6.4 | 0 | 0 | 2.9 |
|  | 157.5 | 350 | 0 | 1029 | 3.5 | 22 | 2496 | 4.2 | 0 | 0 | 4.8 |
|  | 207.9 | 210 | 0 | 953 | 0 | 22 | 2401 | 7.6 | 0 | 0 | 2.1 |
|  | 187.6 | 280 | 0 | 988 | 0 | 22 | 2484 | 5.4 | 0 | 0 | 3 |
|  | 150.5 | 350 | 0 | 982 | 3.5 | 22 | 2363 | 3.6 | 0 | 0 | 4.9 |
|  | 205.8 | 210 | 0 | 977 | 0 | 22 | 2447 | 6.9 | 0 | 0 | 2.2 |
|  | 190.4 | 280 | 0 | 962 | 0 | 22 | 2458 | 5.8 | 0 | 0 | 3 |
|  | 157.5 | 350 | 0 | 1016 | 3.5 | 22 | 2464 | 3.9 | 0 | 0 | 5.0 |
| 17 | 179 | 275 | 735 | 184 | 0 | 19 | 2320 | 5.3 | 0 | 0 | 4.1 |
|  | 179 | 275 | 455 | 455 | 0 | 19 | 2320 | 5.3 | 0 | 0 | 4.7 |
|  | 179 | 275 | 0 | 830 | 0 | 19 | 2320 | 5.3 | 0 | 0 | 4.9 |
|  | 190 | 380 | 757 | 189 | 0 | 19 | 2320 | 5.3 | 0 | 0 | 4.7 |
|  | 190 | 380 | 471 | 471 | 0 | 19 | 2320 | 5.3 | 0 | 0 | 4.8 |
|  | 190 | 380 | 0 | 874 | 0 | 19 | 2320 | 5.3 | 0 | 0 | 5.0 |
|  | 179 | 275 | 740 | 185 | 0 | 19 | 2320 | 5.3 | 0 | 0 | 2.5 |
|  | 179 | 275 | 408 | 408 | 0 | 19 | 2320 | 5.3 | 0 | 0 | 2.4 |
|  | 179 | 275 | 0 | 640 | 0 | 19 | 2320 | 5.3 | 0 | 0 | 2.3 |
|  | 190 | 380 | 767 | 192 | 0 | 19 | 2320 | 5.3 | 0 | 0 | 3.2 |
|  | 190 | 380 | 426 | 427 | 0 | 19 | 2320 | 5.3 | 0 | 0 | 2.7 |
|  | 190 | 380 | 0 | 683 | 0 | 19 | 2320 | 5.3 | 0 | 0 | 2.4 |
|  | 179 | 325 | 839 | 210 | 0 | 19 | 2320 | 5.3 | 0 | 0 | 2.9 |
|  | 179 | 325 | 490 | 490 | 0 | 19 | 2320 | 5.3 | 0 | 0 | 2.6 |
|  | 179 | 325 | 0 | 923 | 0 | 19 | 2320 | 5.3 | 0 | 0 | 2.4 |
|  | 173 | 385 | 892 | 233 | 0 | 19 | 2320 | 5.3 | 0 | 0 | 3.5 |
|  | 173 | 385 | 515 | 515 | 0 | 19 | 2320 | 5.3 | 0 | 0 | 2.9 |
|  | 173 | 385 | 0 | 963 | 0 | 19 | 2320 | 5.3 | 0 | 0 | 2.5 |
| 18 | 227 | 412.7 | 490 | 437.5 | 0 | 12.5 | 2390 | 8.64 | 0 | 0 | 2.68 |
|  | 227 | 412.7 | 490 | 437.5 | 0.89 | 12.5 | 2390 | 8.64 | 0.25 | 5 | 3.13 |
|  | 227 | 412.7 | 490 | 437.5 | 1.45 | 12.5 | 2390 | 8.64 | 0.5 | 5 | 3.2 |
|  | 227 | 412.7 | 490 | 437.5 | 2.31 | 12.5 | 2390 | 8.64 | 0.75 | 5 | 3.18 |
|  | 227 | 412.7 | 490 | 437.5 | 2.84 | 12.5 | 2390 | 8.64 | 1 | 5 | 3.17 |
|  | 259 | 470.1 | 0 | 875 | 0 | 12.5 | 2390 | 8.64 | 0 | 0 | 2.5 |
|  | 259 | 470.1 | 0 | 875 | 0.89 | 12.5 | 2390 | 8.64 | 0.25 | 5 | 2.87 |
|  | 259 | 470.1 | 0 | 875 | 1.45 | 12.5 | 2390 | 8.64 | 0.5 | 5 | 2.99 |
|  | 259 | 470.1 | 0 | 875 | 2.31 | 12.5 | 2390 | 8.64 | 0.75 | 5 | 3 |
|  | 259 | 470.1 | 0 | 875 | 2.84 | 12.5 | 2390 | 8.64 | 1 | 5 | 3.02 |
| 19 | 192 | 426 | 0 | 905 | 1.34 | 20 | 2240 | 5.53 | 0 | 0 | 2.62 |
|  | 192 | 426 | 0 | 905 | 1.34 | 20 | 2240 | 5.53 | 0.5 | 3 | 2.94 |
|  | 192 | 426 | 0 | 905 | 1.34 | 20 | 2240 | 5.53 | 0.75 | 3 | 2.65 |
|  | 192 | 426 | 0 | 905 | 1.34 | 20 | 2240 | 5.53 | 1 | 3 | 2.57 |
| 20 | 180.5 | 340.6 | 609.6 | 609.6 | 0 | 19 | 2172 | 2.25 | 0 | 0 | 2.4 |
|  | 180.5 | 340.6 | 0 | 1186.4 | 0 | 19 | 2172 | 2.25 | 0 | 0 | 1.8 |
|  | 180.5 | 340.6 | 609.6 | 609.6 | 0 | 19 | 2172 | 2.25 | 077 | 4 | 1.6 |
|  | 180.5 | 340.6 | 0 | 1186.4 | 0 | 19 | 2172 | 2.25 | 077 | 4 | 1.5 |
|  | 180.5 | 340.6 | 609.6 | 609.6 | 0 | 19 | 2172 | 2.25 | 0.144 | 4 | 1.9 |
|  | 180.5 | 340.6 | 0 | 1186.4 | 0 | 19 | 2172 | 2.25 | 0.144 | 4 | 1.7 |
| 21 | 205 | 337 | 576 | 576 | 2.2 | 20 | 2491 | 3.4 | 0.1 | 4 | 3.89 |
|  | 205 | 337 | 576 | 576 | 2.2 | 20 | 2491 | 3.4 | 0.136 | 2 | 4.18 |
|  | 205 | 337 | 576 | 576 | 2.2 | 20 | 2491 | 3.4 | 0.1 | 1 | 4 |
|  | 205 | 337 | 576 | 576 | 2.2 | 20 | 2491 | 3.4 | 0.1 | 3 | 4.22 |
|  | 205 | 337 | 576 | 576 | 2.2 | 20 | 2491 | 3.4 | 05 | 4 | 3.53 |
|  | 205 | 337 | 576 | 576 | 2.2 | 20 | 2491 | 3.4 | 0.1 | 4 | 3.89 |
|  | 205 | 337 | 576 | 576 | 2.2 | 20 | 2491 | 3.4 | 0.2 | 4 | 3.9 |
| 22 | 170 | 314.8 | 534 | 534 | 3.2 | 20 | 2640 | 4.85 | 1 | 1 | 4.79 |
|  | 170 | 435.9 | 506 | 506 | 4.4 | 20 | 2640 | 4.85 | 1 | 1 | 5.57 |
|  | 170 | 548.4 | 476 | 476 | 5.5 | 20 | 2640 | 4.85 | 1 | 1 | 6.72 |
|  | 169 | 422.5 | 735 | 315 | 4.2 | 20 | 2640 | 4.85 | 1 | 1 | 5.53 |
|  | 175 | 473 | 0 | 938 | 4.7 | 20 | 2640 | 4.85 | 1 | 1 | 5.35 |
|  | 158 | 405 | 553 | 553 | 4.1 | 20 | 2640 | 4.85 | 0 | 0 | 2.77 |
|  | 164 | 420 | 527 | 527 | 4.2 | 20 | 2640 | 4.85 | 0.5 | 1 | 4.11 |
|  | 177 | 453.8 | 506 | 506 | 4.5 | 20 | 2640 | 4.85 | 1.5 | 1 | 7.02 |
|  | 183 | 469.2 | 473 | 473 | 4.7 | 20 | 2640 | 4.85 | 2 | 1 | 7.61 |
| 23 | 200 | 400 | 720 | 240 | 1.18 | 12.5 | 2190 | 5.64 | 0 | 0 | 2.62 |
|  | 200 | 400 | 480 | 480 | 1.9 | 12.5 | 2190 | 5.64 | 0 | 0 | 2.54 |
|  | 200 | 400 | 240 | 720 | 2.05 | 12.5 | 2190 | 5.64 | 0 | 0 | 2.41 |
|  | 200 | 400 | 240 | 720 | 2.15 | 12.5 | 2190 | 5.64 | 0 | 0 | 2.35 |
|  | 200 | 400 | 720 | 240 | 2.23 | 12.5 | 2190 | 5.64 | 025 | 3 | 2.85 |
|  | 200 | 400 | 480 | 480 | 2.38 | 12.5 | 2190 | 5.64 | 005 | 3 | 2.96 |
|  | 200 | 400 | 240 | 720 | 2.45 | 12.5 | 2190 | 5.64 | 0.1 | 3 | 3.11 |
|  | 200 | 400 | 0 | 960 | 2.5 | 12.5 | 2190 | 5.64 | 0.15 | 3 | 2.82 |
| 24 | 343.5 | 514.5 | 824.4 | 206.1 | 0 | 19 | 2580 | 5.2 | 0 | 0 | 2.34 |
|  | 343.5 | 514.5 | 824.4 | 206.1 | 0 | 19 | 2580 | 5.2 | 0.1 | 4 | 2.42 |
|  | 343.5 | 514.5 | 824.4 | 206.1 | 0 | 19 | 2580 | 5.2 | 0.3 | 4 | 2.63 |
|  | 343.5 | 514.5 | 824.4 | 206.1 | 0 | 19 | 2580 | 5.2 | 0.5 | 4 | 2.81 |
|  | 343.5 | 514.5 | 824.4 | 206.1 | 0 | 19 | 2580 | 5.2 | 1 | 4 | 2.93 |
|  | 343.5 | 514.5 | 824.4 | 206.1 | 0 | 19 | 2580 | 5.2 | 1.5 | 4 | 3.28 |
| 25 | 190 | 378 | 0 | 1052 | 2.16 | 20 | 2600 | 3.8 | 0.1 | 4 | 2.91 |
|  | 190 | 378 | 0 | 1052 | 2.16 | 20 | 2600 | 3.8 | 0.2 | 4 | 3.15 |
|  | 190 | 378 | 0 | 1052 | 2.16 | 20 | 2600 | 3.8 | 0.3 | 4 | 3.07 |
|  | 190 | 378 | 0 | 1052 | 2.16 | 20 | 2600 | 3.8 | 0.4 | 4 | 2.98 |
|  | 163 | 362 | 0 | 1099 | 1.81 | 20 | 2600 | 3.8 | 0.1 | 4 | 3.39 |
|  | 163 | 362 | 0 | 1099 | 1.81 | 20 | 2600 | 3.8 | 0.2 | 4 | 3.57 |
|  | 163 | 362 | 0 | 1099 | 1.81 | 20 | 2600 | 3.8 | 0.3 | 4 | 3.61 |
|  | 163 | 362 | 0 | 1099 | 1.81 | 20 | 2600 | 3.8 | 0.4 | 4 | 3.43 |
|  | 160 | 414 | 0 | 1112 | 2.21 | 20 | 2600 | 3.8 | 0.1 | 4 | 3.95 |
|  | 160 | 414 | 0 | 1112 | 2.21 | 20 | 2600 | 3.8 | 0.2 | 4 | 3.64 |
|  | 160 | 414 | 0 | 1112 | 2.21 | 20 | 2600 | 3.8 | 0.3 | 4 | 4.04 |
|  | 160 | 414 | 0 | 1112 | 2.21 | 20 | 2600 | 3.8 | 0.4 | 4 | 4.14 |
| 26 | 184.5 | 410 | 605 | 605 | 0.5 | 20 | 2480 | 4.14 | 0.16 | 5 | 3.4 |
|  | 184.5 | 410 | 605 | 605 | 0.5 | 20 | 2480 | 4.14 | 0.19 | 6 | 3.2 |
|  | 184.5 | 410 | 605 | 605 | 0.5 | 20 | 2480 | 4.14 | 0.4 | 1 | 3.9 |
| 27 | 210 | 425 | 590 | 529 | 0 | 20 | 2460 | 6.07 | 0.4 | 4 | 1.92 |
|  | 210 | 425 | 0 | 1058 | 0 | 20 | 2460 | 6.07 | 0.4 | 4 | 1.71 |
|  | 210 | 425 | 590 | 529 | 0 | 20 | 2460 | 6.07 | 0.75 | 4 | 1.96 |
|  | 210 | 425 | 0 | 1058 | 0 | 20 | 2460 | 6.07 | 0.75 | 4 | 1.7 |
|  | 210 | 425 | 590 | 529 | 0 | 20 | 2460 | 6.07 | 1.13 | 4 | 1.91 |
|  | 210 | 425 | 0 | 1058 | 0 | 20 | 2460 | 6.07 | 1.13 | 4 | 1.65 |
|  | 210 | 412.5 | 590 | 529 | 0 | 20 | 2460 | 6.07 | 0.4 | 4 | 2.01 |
|  | 210 | 412.5 | 0 | 1058 | 0 | 20 | 2460 | 6.07 | 0.4 | 4 | 1.43 |
|  | 210 | 412.5 | 590 | 529 | 0 | 20 | 2460 | 6.07 | 0.75 | 4 | 2.03 |
|  | 210 | 412.5 | 0 | 1058 | 0 | 20 | 2460 | 6.07 | 0.75 | 4 | 1.46 |
|  | 210 | 412.5 | 590 | 529 | 0 | 20 | 2460 | 6.07 | 1.13 | 4 | 2 |
|  | 210 | 412.5 | 0 | 1058 | 0 | 20 | 2460 | 6.07 | 1.13 | 4 | 1.66 |
|  | 210 | 400 | 590 | 529 | 0 | 20 | 2460 | 6.07 | 0.4 | 4 | 1.85 |
|  | 210 | 400 | 0 | 1058 | 0 | 20 | 2460 | 6.07 | 0.4 | 4 | 1.76 |
|  | 210 | 400 | 590 | 529 | 0 | 20 | 2460 | 6.07 | 0.75 | 4 | 1.9 |
|  | 210 | 400 | 0 | 1058 | 0 | 20 | 2460 | 6.07 | 0.75 | 4 | 1.49 |
|  | 210 | 400 | 590 | 529 | 0 | 20 | 2460 | 6.07 | 1.13 | 4 | 1.98 |
|  | 210 | 400 | 0 | 1058 | 0 | 20 | 2460 | 6.07 | 1.13 | 4 | 1.51 |
|  | 210 | 391 | 590 | 529 | 0 | 20 | 2460 | 6.07 | 0.4 | 4 | 1.96 |
|  | 210 | 391 | 0 | 1058 | 0 | 20 | 2460 | 6.07 | 0.4 | 4 | 1.56 |
|  | 210 | 391 | 590 | 529 | 0 | 20 | 2460 | 6.07 | 0.75 | 4 | 1.94 |
|  | 210 | 391 | 0 | 1058 | 0 | 20 | 2460 | 6.07 | 0.75 | 4 | 1.68 |
|  | 210 | 391 | 590 | 529 | 0 | 20 | 2460 | 6.07 | 1.13 | 4 | 1.83 |
|  | 210 | 391 | 0 | 1058 | 0 | 20 | 2460 | 6.07 | 1.13 | 4 | 1.65 |
| 28 | 137.5 | 327.6 | 0 | 1474 | 0 | 19 | 2610 | 5.73 | 0.1 | 1 | 1.78 |
|  | 137.5 | 327.6 | 0 | 1474 | 0 | 19 | 2610 | 5.73 | 0.15 | 1 | 1.84 |
|  | 137.5 | 327.6 | 0 | 1474 | 0 | 19 | 2610 | 5.73 | 0.2 | 1 | 1.7 |
|  | 137.5 | 327.6 | 0 | 1474 | 0 | 19 | 2610 | 5.73 | 0.25 | 1 | 1.89 |
|  | 137.5 | 327.6 | 0 | 1474 | 0 | 19 | 2610 | 5.73 | 0.3 | 1 | 2.33 |
|  | 137.5 | 327.6 | 0 | 1474 | 0 | 19 | 2610 | 5.73 | 0.2 | 1 | 2.15 |
|  | 137.5 | 327.6 | 0 | 1474 | 0 | 19 | 2610 | 5.73 | 0.3 | 1 | 2.51 |
|  | 137.5 | 327.6 | 0 | 1474 | 0 | 19 | 2610 | 5.73 | 0.4 | 1 | 2.38 |
|  | 137.5 | 327.6 | 0 | 1474 | 0 | 19 | 2610 | 5.73 | 0.5 | 1 | 2.14 |
|  | 137.5 | 327.6 | 0 | 1474 | 0 | 19 | 2610 | 5.73 | 0.6 | 1 | 2.25 |
|  | 137.5 | 327.6 | 0 | 1474 | 0 | 19 | 2610 | 5.73 | 0.3 | 1 | 1.72 |
|  | 137.5 | 327.6 | 0 | 1474 | 0 | 19 | 2610 | 5.73 | 0.45 | 1 | 1.64 |
|  | 137.5 | 327.6 | 0 | 1474 | 0 | 19 | 2610 | 5.73 | 0.6 | 1 | 1.7 |
|  | 137.5 | 327.6 | 0 | 1474 | 0 | 19 | 2610 | 5.73 | 0.75 | 1 | 1.75 |
|  | 137.5 | 327.6 | 0 | 1474 | 0 | 19 | 2610 | 5.73 | 0.9 | 1 | 1.68 |
| 29 | 98.28 | 327.59 | 0 | 1474 | 0 | 20 | 2609 | 4.83 | 0.3 | 3 | 1.4 |
|  | 98.28 | 327.59 | 0 | 1474 | 0 | 20 | 2609 | 4.83 | 0.6 | 3 | 2 |
|  | 98.28 | 327.59 | 0 | 1474 | 0 | 20 | 2609 | 4.83 | 0.9 | 3 | 1.77 |
|  | 98.28 | 327.59 | 0 | 1474 | 0 | 20 | 2609 | 4.83 | 1.2 | 3 | 1.38 |
|  | 98.28 | 327.59 | 0 | 1474 | 0 | 20 | 2609 | 4.83 | 0.2 | 2 | 1.66 |
|  | 98.28 | 327.59 | 0 | 1474 | 0 | 20 | 2609 | 4.83 | 0.4 | 2 | 1.77 |
|  | 98.28 | 327.59 | 0 | 1474 | 0 | 20 | 2609 | 4.83 | 0.6 | 2 | 1.59 |
|  | 98.28 | 327.59 | 0 | 1474 | 0 | 20 | 2609 | 4.83 | 0.8 | 2 | 1.46 |
| 30 | 180 | 400 | 850.8 | 364.7 | 0 | 25 | 2563 | 4.92 | 0.6 | 1 | 2.68 |
|  | 180 | 400 | 607.8 | 607.8 | 0 | 25 | 2563 | 4.92 | 1.2 | 1 | 3.8 |
|  | 180 | 400 | 0 | 1215.5 | 0 | 25 | 2563 | 4.92 | 1.8 | 1 | 4.64 |
|  | 180 | 400 | 850.8 | 364.7 | 0 | 25 | 2563 | 4.92 | 0 | 0 | 2.77 |
|  | 180 | 360 | 0 | 1215.5 | 0 | 25 | 2563 | 4.92 | 0.6 | 1 | 3.62 |
|  | 180 | 360 | 607.8 | 607.8 | 0 | 25 | 2563 | 4.92 | 1.8 | 1 | 4.57 |
|  | 180 | 320 | 607.8 | 607.8 | 0 | 25 | 2563 | 4.92 | 0 | 0 | 2.47 |
|  | 180 | 320 | 0 | 1215.5 | 0 | 25 | 2563 | 4.92 | 0.6 | 1 | 3.06 |
|  | 180 | 320 | 850.8 | 364.7 | 0 | 25 | 2563 | 4.92 | 1.8 | 1 | 3.91 |
|  | 180 | 280 | 0 | 1215.5 | 0 | 25 | 2563 | 4.92 | 0 | 0 | 2.62 |
|  | 180 | 280 | 607.8 | 607.8 | 0 | 25 | 2563 | 4.92 | 0.6 | 1 | 2.74 |
|  | 180 | 280 | 850.8 | 364.7 | 0 | 25 | 2563 | 4.92 | 1.2 | 1 | 3.06 |

For fiber type, 1-Steel fiber, 2-Carbon fiber, 3- Polypropylene fiber, 4-Basalt fiber, 5-Glass fiber, 6-Woolen fiber.

**References**

1 Gómez-Soberón J M V. Porosity of recycled concrete with substitution of recycled concrete aggregate: An experimental study. Cement and concrete research, 2002, 32(8): 1301-1311.

2 Etxeberria M, Marí A R, Vázquez E. Recycled aggregate concrete as structural material. Materials and structures, 2007, 40(5): 529-541.

3 Kou S C, Poon C S, Chan D. Influence of fly ash as cement replacement on the properties of recycled aggregate concrete. Journal of materials in civil engineering, 2007, 19(9): 709-717.

4 Kou S C, Poon C S, Chan D. Influence of fly ash as a cement addition on the hardened properties of recycled aggregate concrete. Materials and Structures, 2008, 41(7): 1191-1201.

5 Yang K H, Chung H S, Ashour A F. Influence of type and replacement level of recycled aggregates on concrete properties. Aci Materials Journal, 2008, 105(3):289-296.

6 Zega C J, Di Maio A A. Recycled concretes made with waste ready-mix concrete as coarse aggregate. Journal of Materials in Civil Engineering, 2011, 23(3): 281-286.

7 Fathifazl G, Razaqpur A G, Isgor O B, et al. Creep and drying shrinkage characteristics of concrete produced with coarse recycled concrete aggregate. Cement & Concrete Composites, 2011, 33(10):1026-1037.

8 Chakradhara Rao M, Bhattacharyya S K, Barai S V. Influence of field recycled coarse aggregate on properties of concrete. Materials and structures, 2011, 44(1): 205-220.

9 Pereira P, Evangelista L, De Brito J. The effect of superplasticizers on the mechanical performance of concrete made with fine recycled concrete aggregates. Cement and concrete composites, 2012, 34(9): 1044-1052.

10 Butler L, West J S, Tighe S L. Effect of recycled concrete coarse aggregate from multiple sources on the hardened properties of concrete with equivalent compressive strength. Construction and Building Materials, 2013, 47: 1292-1301.

11 Thomas C, Setién J, Polanco J A, et al. Durability of recycled aggregate concrete. Construction and building materials, 2013, 40: 1054-1065.

12 Younis K H, Pilakoutas K. Strength prediction model and methods for improving recycled aggregate concrete. Construction and Building Materials, 2013, 49: 688-701.

13 Andreu G, Miren E. Experimental analysis of properties of high performance recycled aggregate concrete. Construction and Building Materials, 2014, 52: 227-235.

14 Duan Z H, Poon C S. Properties of recycled aggregate concrete made with recycled aggregates with different amounts of old adhered mortars. Materials & Design, 2014, 58: 19-29.

15 Folino P, Xargay H. Recycled aggregate concrete–Mechanical behavior under uniaxial and triaxial compression. Construction and Building Materials, 2014, 56: 21-31.

16 Pedro D, De Brito J, Evangelista L. Performance of concrete made with aggregates recycled from precasting industry waste: influence of the crushing process. Materials and Structures, 2015, 48(12): 3965-3978.

17 Thomas C, Sosa I, Setién J, et al. Evaluation of the fatigue behavior of recycled aggregate concrete. Journal of Cleaner Production, 2014, 65: 397-405.

18 Ali B, Qureshi L A. Influence of glass fibers on mechanical and durability performance of concrete with recycled aggregates. Construction and Building Materials, 2019, 228: 116783.

19 Das C S, Dey T, Dandapat R, et al. Performance evaluation of polypropylene fibre reinforced recycled aggregate concrete. Construction and Building Materials, 2018, 189: 649-659.

20 Dong J F, Wang Q Y, Guan Z W. Material properties of basalt fibre reinforced concrete made with recycled earthquake waste. Construction and Building Materials, 2017, 130: 241-251.

21 Fang S E, Hong H S, Zhang P H. Mechanical property tests and strength formulas of basalt fiber reinforced recycled aggregate concrete. Materials, 2018, 11(10): 1851.

22 Gao D, Zhang L. Flexural performance and evaluation method of steel fiber reinforced recycled coarse aggregate concrete. Construction and Building Materials, 2018, 159: 126-136.

23 Ibrahm H A, Abbas B J. Mechanical behavior of recycled self-compacting concrete reinforced with polypropylene fibres. Journal of Architectural Engineering Technology, 2017, 6(2): 1-7.

24 Katkhuda H, Shatarat N. Improving the mechanical properties of recycled concrete aggregate using chopped basalt fibers and acid treatment. Construction and Building Materials, 2017, 140: 328-335.

25 Liu H, Yang J, Kong X, et al. Basic mechanical properties of basalt fiber reinforced recycled aggregate concrete. The open civil engineering journal, 2017, 11(1).

26 Meesala C R. Influence of different types of fiber on the properties of recycled aggregate concrete. Structural Concrete, 2019, 20(5): 1656-1669.

27 Wang Y, Hughes P, Niu H, et al. A new method to improve the properties of recycled aggregate concrete: Composite addition of basalt fiber and nano-silica. Journal of Cleaner Production, 2019, 236: 117602.

28 Chen S, Lu P, Li B, et al. The evaluation of influence of different fibers on the properties of recycled aggregate pervious concrete. Journal of Basic Science and Engineering, 2022, 30(1): 208-218. (In Chinese)

29 Zhang X, Kuang C, Fang Z, et al. Orthogonal experimental study on strength of steel fiber reinforced fly ash recycled concrete. Journal of Building Materials, 2014,17: 677-694. (In Chinese)

30 Guo L, Liu S, Chen S, et al. Study on mechanical properties, water permeability and wear resistance of fiber modified recycled aggregate pervious concrete. Transactions of the Chinese Society of Agricultural Engineering, 2019,35(02):153-160. (In Chinese)
